# Functional Fitness Deficits in Women with Fibromyalgia Relative to Later-Life Independence Standards: Contributions of Sedentary Time and Physical Activity

**DOI:** 10.3390/sports14080322

**Published:** 2026-08-01

**Authors:** Cristina Maestre-Cascales, Pedro Acosta-Manzano, Ciara M. Hughes, Víctor Segura-Jiménez, Blanca Gavilán-Carrera

**Affiliations:** 1 LFE Research Group, Department of Health and Human Performance, Faculty of Physical Activity and Sport Sciences, Universidad Politécnica de Madrid, 28040 Madrid, Spain; 2 Department of Human Movement Science, Sport and Health, University of Graz, 8010 Graz, Austria; pedro.acosta-manzano@uni-graz.at; 3 Institute of Nursing and Health Research, School of Health Sciences, Ulster University, Londonderry BT48 7JL, UK; cm.hughes@ulster.ac.uk; 4 UGC Medicina Física y Rehabilitación, Hospital Universitario Virgen de las Nieves, 18013 Granada, Spain; vsegura@ibsgranada.es; 5 Instituto de Investigación Biosanitaria ibs.GRANADA, 18012 Granada, Spain; 6 GALENO Research Group, Department of Physical Education, Faculty of Education Sciences, University of Cádiz, 11519 Puerto Real, Spain; 7 Instituto de Investigación e Innovación Biomédica de Cádiz (INiBICA), 11009 Cádiz, Spain; 8 PA-HELP “Physical Activity for HEaLth Promotion” Research Group, Sport and Health University Research Institute (iMUDS), University of Granada, 18071 Granada, Spain; 9 Department of Physical Education and Sports, Faculty of Sports Science, University of Granada, 18071 Granada, Spain

**Keywords:** fibromyalgia, aerobic capacity, muscular strength, physical activity, sedentary behaviour, accelerometry, healthy ageing, functional independence, women

## Abstract

Background: Maintaining adequate physical fitness is essential for preserving functional independence with ageing. Women with fibromyalgia often exhibit reduced fitness levels, and daily sedentary time (ST) and physical activity (PA) may be important contributors to physical fitness. Objectives: To examine (1) the proportion of women with fibromyalgia meeting fitness standards for later-life independence, (2) associations of ST and PA with fitness, and (3) how reallocating ST to PA relates to fitness. Methods: 407 women with fibromyalgia (51.9 ± 8.0 years) participated. Fitness (Senior Fitness Test and handgrip strength) was compared with standards for women aged 60–64 as indicators of later-life independence. ST and PA were measured using triaxial accelerometry. Linear and isotemporal substitution models examined associations and estimated the theoretical associations of replacing ST with light, moderate, or vigorous PA in relation to physical fitness. Results: 13.3% of participants met cardiorespiratory, 7.9% lower-body strength, 29.9% upper-body endurance, and 4.4% agility standards. ST and PA were associated with fitness (all *p* < 0.05; adj R^2^ < 0.06). Reallocating 30 min of ST to light PA was associated with higher handgrip strength (B = 0.3); with moderate PA, cardiorespiratory fitness (B = 9.0); and with vigorous PA, upper-body muscular endurance (B = 11.1) and cardiorespiratory fitness (B = 104.9), all *p* < 0.05. Conclusions: Overall, only a small proportion of women met established fitness reference standards for later-life independence. ST was negatively and PA positively associated with fitness, although these associations explained little of its variability. In isotemporal substitution models, estimated replacement of ST with PA was associated with better fitness outcomes, particularly cardiorespiratory fitness and upper-body muscular endurance, with stronger associations at higher PA intensities. Further research is needed to identify additional modifiable determinants of physical fitness and support long-term functional independence.

## 1. Introduction

Fibromyalgia is a chronic condition characterized by widespread pain, fatigue, and reduced physical and psychological well-being [1]. Its impact on physical health is particularly pronounced [2], with individuals showing marked impairments in cardiorespiratory fitness, muscular strength, flexibility and agility [3]. In the general population, physical fitness is recognized as a powerful marker of health and a key determinant of independence in later life [4]. Among individuals with fibromyalgia, lower physical fitness is further associated with more severe symptomatology [5]. Therefore, given the substantial fitness deficits in this population, assessing the risk of future loss of independence and identifying modifiable fitness determinants is essential to preserve health and autonomy.

Daily physical activity (PA) is a key determinant of physical fitness. Promoting PA in fibromyalgia is challenging, and evidence on effective interventions is limited [6]. Previous studies, mostly based on self-reported PA, have shown minimal or short-term improvements in physical fitness following PA interventions [6]. Increasing light PA alone may be insufficient to improve fitness unless baseline activity levels are very low [7], as is often the case in women with fibromyalgia [3]. Higher-intensity activities, such as moderate-to-vigorous PA, are likely more beneficial for health and fitness [7]. However, the relative contribution of different PA intensities to physical fitness has not been well established in this population. In addition to PA, sedentary time (ST) has emerged as an independent movement behaviour that may also influence physical fitness [8].

Sedentary time (ST) is defined as any waking behaviour performed while sitting, reclining or lying with an energy expenditure ≤1.5 metabolic equivalents (e.g., sitting at work, television viewing, reading, or other screen-based activities) [9]. ST is conceptually distinct from physical inactivity, as individuals may achieve the recommended levels of PA while still accumulating excessive ST [8]. Evidence indicates that ST is associated with poorer physical and mental health, including reduced physical function, lower quality of life, and adverse cardiometabolic outcomes [10]. Importantly, ST is also inversely associated with physical fitness [11], highlighting the importance of examining ST in relation to fitness outcomes. Women with fibromyalgia typically spend large amounts of time sedentary, partly because pain, fatigue, fear of movement (kinesiophobia), and concerns about symptom exacerbation discourage engagement in PA [12,13]. This behavioural pattern may contribute to a vicious cycle in which these factors and ST reinforce one another, potentially leading to physical deconditioning and poorer physical fitness [12,13]. However, the relationship between ST and physical fitness in women with fibromyalgia remains poorly understood.

Because time spent in different movement behaviours is finite, increasing time in one behaviour necessarily requires reducing time in another. Isotemporal substitution modelling estimates the associations of replacing time spent in one behaviour (e.g., ST) with an equal amount of another (e.g., light, moderate, or vigorous PA), while keeping total time constant [14]. Compared with analyzing each behaviour independently, this approach better reflects the co-dependent nature of movement behaviours and provides a more meaningful framework for examining their associations with physical fitness. However, whether isotemporal reallocation of ST to different PA intensities is associated with physical fitness in women with fibromyalgia remains unknown. Exploring these associations may generate hypotheses to inform future longitudinal and intervention studies in women with fibromyalgia. Therefore, this exploratory study aimed to: (i) determine the proportion of women with fibromyalgia meeting fitness standards for maintaining independence in later life; (ii) examine the associations of ST and PA with physical fitness; and (iii) estimate the theoretical associations of reallocating ST to light, moderate, or vigorous PA with physical fitness.

## 2. Materials and Methods

### 2.1. Study Sample and Design

Participants were mainly recruited from southern Spanish fibromyalgia associations and provided written informed consent after receiving study information. Inclusion criteria were: (i) women aged 18–65 years, (ii) meeting the 1990 ACR fibromyalgia criteria [15], (iii) absence of acute or terminal illness, and (iv) no severe cognitive impairment. The study was approved by the Ethics Committee of Hospital Virgen de las Nieves, Granada, Spain.

### 2.2. Procedure

Assessments were conducted over two appointments by the same researcher. At the first visit, fibromyalgia diagnosis was confirmed and height and weight were measured. Two days later, physical fitness was evaluated and participants received an accelerometer to wear for nine consecutive days.

### 2.3. Materials

#### 2.3.1. Fibromyalgia Diagnosis

Using a standard pressure algometer (FPK 20, Effegi, Alfonsine, Italy), 18 tender points were assessed per the 1990 ACR criteria [15]. Points were considered positive if pain was reported at ≤4 kg/cm^2^. The average of two measurements per point was used, and fibromyalgia was confirmed if ≥11 points were positive.

#### 2.3.2. Body Mass Index (BMI)

Standing height and body weight were measured following standardized anthropometric procedures [16]. Standing height was assessed using a stadiometer (Seca 22, Hamburg, Germany), and body weight was measured using a portable bioelectrical impedance analyser (InBody R20, Biospace, Seoul, Republic of Korea). BMI was calculated as body weight (kg) divided by standing height squared (m^2^).

#### 2.3.3. Physical Activity and Sedentary Time

Participants wore a triaxial GT3X+ accelerometer (ActiGraph, Pensacola, FL, USA) on the hip for nine days, 24 h/day except during water-based activities. Data were collected at 30 Hz with 60 s epochs [17]. The first (familiarization) and last (device return) days were excluded, leaving seven valid days with ≥10 h/day. Data processing was done using ActiLife™ v6.11.7. Wear time was calculated by subtracting sleep periods identified from sleep onset and wake-up times recorded in sleep diaries. When diary data were incomplete, sleep periods were determined by visual inspection of the accelerometer recordings. Non-wear periods were identified using the Choi algorithm (≥90 min of 0 counts, 30 min small window, 2 min tolerance) [18]. The seven consecutive monitoring days ensured representation of both weekdays and weekend days.

PA intensity was classified using widely adopted vector magnitude cut points [17,18,19]. Although fibromyalgia-specific accelerometer cut points are currently unavailable, these thresholds have been adopted in several previous accelerometer studies of women with fibromyalgia [20,21,22] facilitating comparisons across studies. Specifically, light, moderate, and vigorous PA were defined as 200–2689, 2690–6166, and ≥6167 counts per minute (cpm), respectively [17,19]. ST was estimated as the time accumulated below 200 counts per minute (cpm) during wearing time [18]. All values were converted to 30 min units for easier interpretation by dividing daily minutes spent in each behaviour and total wear time by 30.

#### 2.3.4. Physical Fitness Testing

The Senior Fitness Test battery was chosen for its ability to capture heterogeneity, accommodate the reduced physical capacity of women with fibromyalgia, and for its demonstrated reliability and feasibility [23]. The battery includes: the 30 s chair-stand test, which measures the number of times participants can rise from a seated position to a full stand within 30 s to assess lower-body muscular strength; the arm curl test, which assesses upper-body muscular endurance by recording the number of biceps curls completed with a 2.3 kg dumbbell within 30 s. The test was performed once with each arm, and the average number of repetitions was recorded; the chair sit-and-reach test, which evaluates lower-body flexibility by measuring the distance (cm) between the fingertips and toes while sitting with one leg extended. The test was performed twice for each leg, and the average of the best score from each leg was used; the back-scratch test, which assesses upper-body flexibility by measuring the distance (cm) between the middle fingers behind the back. The test was performed twice, and the average of the best score from each side was used; the 8-foot up-and-go test, in which participants stood up from a chair, walked 2.44 m around a cone, and returned to the seated position as quickly as possible to evaluate agility and dynamic balance. The best time of two trials was recorded; and the 6 min walk test, which measures the maximum distance walked in six minutes along a 45.7 m rectangular course as an indicator of cardiorespiratory fitness [24]. Criterion-referenced standards have been proposed only for certain components, including cardiorespiratory fitness, strength and agility tests to predict the capacity older adults need to maintain independence [4]. Participants also performed the handgrip test, using a digital dynamometer (TKK 5101 Grip-D, Takei, Tokyo, Japan). The test was performed twice with each hand, alternating between hands, and the average of the best score from each hand was used for analysis. Handgrip strength measures maximal isometric strength of the hand and forearm and is commonly used as a marker of upper-body strength in fibromyalgia and the general population [25].

### 2.4. Statistical Analyses

Descriptive statistics summarized sample characteristics, and the proportion meeting criterion-referenced standards for later-life independence was calculated. For the youngest group of older adults (women aged 60–64), thresholds were: 571.5 m in the 6 min walk test, 17 repetitions in the 30 s arm curl test, 15 repetitions in the 30 s chair stand, and 5 s in the agility test [4].

To compare with previous and future research, traditional linear regression models were built as a preliminary step. Linear regression models examined associations of ST and PA intensities with fitness, with each behaviour as the independent variable and each fitness test as the dependent variable. Isotemporal substitution models estimated how reallocating time between behaviours was associated with fitness while keeping total daily time fixed; coefficients represent the association with fitness of replacing 30 min of one behaviour with another. All values were initially expressed in minutes/day but were converted to units of 30 min (1 represents 30 min) for better interpretation of the results. Models included total time and all behaviours except the one being replaced, so each regression coefficient reflects the theoretical effect of substituting the omitted activity with the included activities. For example: performance on the 30 s chair-stand test = β_1_·light PA + β_2_·moderate PA + β_3_·vigorous PA + β_4_·total time + β_5_·covariates. With ST omitted, β_1_ represents the change in chair-stand performance from reallocating 30 min of ST to light PA, and β_2_ reflects the theoretical effect of reallocating 30 min to moderate PA. Age and body mass index were included as covariates in all models. Given the exploratory and hypothesis-generating nature of the analyses, no adjustment for multiple comparisons was applied. Model assumptions were checked (normality, linearity, homoscedasticity, autocorrelation, multicollinearity). Residual diagnostic plots for the models in which vigorous PA was significantly associated with physical fitness are provided in Supplementary Figure S1 to allow evaluation of the regression assumptions in the analyses most likely to be influenced by the highly skewed distribution of vigorous PA. Analyses were conducted in IBM SPSS Statistics for Windows, version 25 (IBM Corp., Armonk, NY, USA), with *p* < 0.05 considered significant.

## 3. Results

Of the 646 individuals who agreed to participate, 154 were excluded for not meeting the study eligibility criteria (39 had not been previously diagnosed by a rheumatologist, 100 did not meet the ACR criteria, 1 had cognitive impairment, and 14 were older than 65 years). Of the remaining 492 eligible participants, 54 were excluded because of accelerometer-related issues and 12 because of incomplete data. Finally, 19 men were excluded, resulting in a final analytical sample of 407 women. Table 1 provides an overview of participants’ characteristics. Overall, 13.3% of participants met the standard for cardiorespiratory fitness, 7.9% for lower-body strength, 29.9% for upper-body muscular endurance, and 4.4% for agility.

Table 2 shows associations of ST and PA intensity with physical fitness from the linear regression models. ST was negatively associated with cardiorespiratory fitness, while light, moderate, and vigorous PA were positively associated with muscular strength, lower-body flexibility, cardiorespiratory fitness, and agility (all *p* < 0.05; adj R^2^ < 0.06). Table 3 presents isotemporal substitution model results. Theoretical reallocation of 30 min of ST to light PA was associated with higher handgrip strength (B = 0.3); to moderate PA, cardiorespiratory fitness (B = 9.0); and to vigorous PA, upper-body endurance (B = 11.1) and cardiorespiratory fitness (B = 104.9), all *p* < 0.05.

## 4. Discussion

Most women with fibromyalgia were below established physical fitness cut-offs associated with premature loss of functional independence. ST was negatively and PA positively associated with fitness outcomes. In isotemporal substitution models, theoretical replacement of ST with PA was associated with better cardiorespiratory fitness and upper-body muscular endurance, particularly at higher PA intensities. However, these associations accounted for only a small proportion of the variability in fitness outcomes.

Our findings indicate that many women with fibromyalgia had physical fitness levels below criterion-referenced thresholds associated with maintaining functional independence in later life. This is especially concerning given that reference values for women aged 60–64 [4] were applied to a sample averaging 51 years. This finding is consistent with previous evidence reporting substantial fitness deficits in other samples of patients with fibromyalgia [26]. Physical fitness is not only an indicator of functional ability but is also associated with the severity of physical and psychological symptoms in fibromyalgia [5]. Indeed, previous studies have shown that fitness assessments can discriminate between different levels of symptom burden and may serve as a complementary tool for disease monitoring [27]. Taken together, these findings highlight the potential value of routine physical fitness assessment as part of the clinical evaluation of women with fibromyalgia.

To our knowledge, no previous studies have examined the associations between objectively measured ST, PA, and fitness in fibromyalgia. Existing evidence in this population suggests that increasing walking can improve self-reported physical function [28], whereas other behaviour-change interventions to increase PA yield mixed results, with some showing no benefits [29] and others improvements in the 6 min walk test [30]. Our findings indicate that higher ST was associated with poorer physical fitness, whereas even light-intensity PA was associated with better performance across multiple fitness domains. Theoretical reallocation of ST to PA was associated with better fitness outcomes, particularly at higher PA intensities. However, high-intensity PA may be less feasible for many people with fibromyalgia and should be introduced gradually. Current guidelines recommend low-to-moderate intensity PA for this population [31], and although high-intensity PA is possible and may provide health benefits among patients [32], it may compromise adherence levels [33].

From a clinical perspective, despite the modest proportion of explained variance, these findings are consistent with the simple and clinically meaningful message of “sit less, move more” for women with fibromyalgia. Although higher-intensity PA showed the strongest associations with physical fitness, encouraging patients to progressively replace ST with any feasible level of PA may represent a practical and achievable approach, particularly for those with pain, fatigue, or low exercise tolerance. However, given the limited variability explained by movement behaviours alone, this message should be considered as one component of a broader, individualized management strategy.

ST and PA explained up to 6% of the variance in fitness components, indicating that although movement behaviours are associated with physical fitness, they account for only a small proportion of its variability. Therefore, fitness in this population seems to be influenced by multiple factors, which could include dysregulated neuromuscular interactions (heightened perceptions of effort, reduced ability to fully activate available musculature, and lower exercise tolerance [26]), symptom management, sleep quality and participation in well-structured exercise programmes. Therefore, promoting daily PA should be considered one component of a comprehensive management approach, together with appropriate symptom management and individualized exercise programmes.

This study has several limitations. First, its cross-sectional design precludes causal inference, although the well-established role of PA in physical fitness is consistent with the observed associations. Second, accelerometry does not capture all activities, such as isolated limb movements or water-based exercise. Third, given the exploratory and hypothesis-generating nature of this study, no correction for multiple comparisons was applied; therefore, the findings should be interpreted conservatively. Fourth, participants were classified according to the 1990 ACR criteria, as prespecified in the original study protocol. As more recent ACR criteria incorporate broader symptom-based assessments [1], the generalizability of our findings to populations classified using these newer criteria may be limited. Finally, vigorous PA was accumulated by only a small proportion of participants, resulting in a highly skewed distribution. Therefore, associations involving vigorous PA should be interpreted conservatively and confirmed in future longitudinal and intervention studies. Strengths of this study include the objective assessment of ST, PA, and physical fitness in a large, representative sample of Andalusian women with fibromyalgia.

## 5. Conclusions

Only a small proportion of women with fibromyalgia met fitness standards for maintaining functional independence in later life. ST was negatively associated with cardiorespiratory fitness, whereas light-, moderate-, and vigorous-intensity PA were positively associated with strength, lower-body flexibility, cardiorespiratory fitness, and agility. However, these associations accounted for only a small proportion of the variability in fitness outcomes. In isotemporal substitution models, theoretical reallocation of 30 min of ST to PA was associated with higher cardiorespiratory fitness and upper-body muscular endurance, with stronger associations observed at higher PA intensities. Further research is needed to identify additional modifiable determinants of physical fitness and to inform more comprehensive strategies to optimize fitness and support long-term functional independence.

## Figures and Tables

**Table 1:** Characteristics of the participants (*n* = 407).

Variables	Mean	SD
Age, years	51.9	8.0
Body mass index (kg/m^2^)	28.6	5.5
Total tender points (11–18)	16.7	2.0
Physical activity and sedentary time (accelerometer, min/day)		
Sedentary time	460.0	103.1
Light physical activity	419.6	91.9
Moderate physical activity	43.6	29.0
Vigorous physical activity	0.4	2.0
Accelerometer-wear time	926.6	75.6
Physical fitness tests		
30 s arm curl test (repetitions)	14.3	4.8
30 s chair stand test (repetitions)	10.4	3.1
Chair sit and reach test (cm)	−11.1	11.5
Back scratch test (cm)	−14.2	12.2
6 min walk test (m)	489.3	76.1
8-foot up and go test ^#^ (s)	6.8	1.7

SD, Standard Deviation. ^#^ Lower scores indicate better performance. For the youngest group of older adults (women aged 60–64), thresholds for functional independence were: 571.5 m in the 6 min walk test, 17 repetitions in the 30 s arm curl test, 15 repetitions in the 30 s chair stand, and 5 s in the agility test [4].

**Table 2:** Individual associations of physical activity (PA) and sedentary time with physical fitness (*n* = 407).

	Sedentary Time				Light PA				Moderate PA				Vigorous PA			
	B	95% CI	Adj R^2^ ST	Adj R^2^ Model	B	95% CI	Adj R^2^ Light PA	Adj R^2^ Model	B	95% CI	Adj R^2^ Mod PA	Adj R^2^ Model	B	95% CI	Adj R^2^ Vigorous PA	Adj R^2^ Model
30 s arm curl test	−0.09	−0.23 to 0.05	<0.001	0.02	0.22 **	0.07 to 0.34	0.01	0.03	0.58 *	0.08 to 1.07	0.01	0.03	12.46 **	5.33 to 19.58	0.03	0.04
Handgrip strength test	−0.14	−0.32 to 0.04	0.01	0.04	0.22 *	0.02 to 0.42	0.01	0.04	−0.24	−0.88 to 0.40	<0.001	0.03	4.19	−5.09 to 13.47	<0.001	0.03
30 s chair stand test	−0.08	−0.17 to 0.01	0.01	0.04	0.15 **	0.05 to 0.25	0.02	0.05	0.42 **	0.11 to 0.72	0.03	0.05	5.59 *	1.12 to 10.08	0.02	0.05
Chair sit and reach test	−0.29	−0.62 to 0.04	0.01	0.02	0.59 **	0.22 to 0.95	0.02	0.03	1.73 **	0.56 to 2.91	0.02	0.03	13.98	−3.11 to 31.08	0.01	0.02
Back scratch test	0.12	−0.21 to 0.44	<0.001	0.16	0.25	−0.11 to 0.61	0.01	0.17	0.79	−0.36 to 1.96	0.02	0.17	4.89	−11.86 to 21.65	0.00	0.16
6 min walk test	−2.32 *	−4.35 to −0.29	0.02	0.16	4.24 ***	2.00 to 6.5	0.04	0.18	13.31 ***	6.15 to 20.48	0.06	0.18	140.65 **	36.27 to 245.03	0.03	0.17
8-foot up-and-go test #	0.03	−0.02 to 0.07	0.01	0.08	−0.07 **	−0.12 to −0.02	0.02	0.10	−0.22 *	−0.38 to −0.05	0.03	0.10	−0.89	−3.29 to 1.49	<0.001	0.08

Coefficients are per 30 min increment. B, unstandardized regression coefficient. CI, Confidence interval. * *p* < 0.05; ** *p* < 0.01; *** *p* < 0.001. All the models included body mass index and age as covariates. # Lower scores indicate better performance.

**Table 3:** Associations of reallocating 30 min of sedentary time to 30 min of light, moderate or vigorous physical activity (PA) with physical fitness components (*n* = 407).

	Light PA				Moderate PA				Vigorous PA			
	B	95% CI		*p*	B	95% CI		*p*	B	95% CI		*p*
30 s arm curl test	0.11	−0.06	0.29	0.210	0.28	−0.25	0.80	0.299	**11.09**	**3.89**	**18.28**	**0.003**
30 s chair stand test	0.09	−0.02	0.20	0.125	0.25	−0.08	0.58	0.140	4.51	0.00	9.02	0.050
Chair sit and reach test	0.32	−0.10	0.74	0.138	1.20	−0.05	2.45	0.060	9.11	−8.05	26.27	0.297
Back scratch test	−0.07	−0.49	0.34	0.730	0.68	−0.55	1.92	0.277	2.46	−14.45	19.36	0.775
6 min walk test	2.31	−0.26	4.88	0.078	**9.02**	**1.43**	**16.61**	**0.020**	**104.85**	**0.75**	**208.94**	**0.048**
8-foot up and go test #	−0.03	−0.09	0.03	0.304	−0.17	−0.34	0.01	0.064	−0.25	−2.66	2.15	0.837
Handgrip strength test	**0.31**	**0.08**	**0.54**	**0.009**	−0.60	−1.28	0.09	0.089	4.82	−4.57	14.20	0.314

B, non-standardized regression coefficient. CI, Confidence interval. All the models included body mass index, and age, and total wear time (sedentary time + light PA + moderate PA + vigorous PA). Coefficients are expressed per 30 min/day reallocation; bold values indicate statistically significant associations (*p* < 0.05). # Lower scores indicate better performance.

## Data Availability

The datasets generated and/or analyzed during the current study are available from the corresponding authors upon reasonable request.

## Supplementary Materials

The following supporting information can be downloaded at: https://www.mdpi.com/article/10.3390/sports14080322/s1, Supplementary Figure S1. Residual diagnostic plots for the models in which vigorous physical activity was significantly associated with physical fitness.

## Abbreviations

The following abbreviations are used in this manuscript:

ACR	American College of Rheumatology
BMI	Body Mass Index
PA	Physical Activity
ST	Sedentary Time
