# Supplementary material for: Functional Fitness Deficits in Women with Fibromyalgia Relative to Later-Life Independence Standards: Contributions of Sedentary Time and Physical Activity
Source: Sports (Basel). 2026 Aug 1;14(8):322. doi: 10.3390/sports14080322 (PMC13517400; doi:10.3390/sports14080322)
Supplement: Supplementary file 1 [file sports-14-00322-s001.zip › sports-4388798-supplementary.pdf]

Supplementary Figure S1. Residual diagnostic plots for the models in which vigorous physical activity was significantly associated with physical fitness

	**30-s Arm curl**<br>Individual Model	**6-min Walk**<br>Individual Model	**30-s Chair stand**<br>Individual Model	**6-min Walk**<br>Isotemporal Model	**30-s Arm-curl**<br>Isotemporal Model
**Histogram of standardized residuals**	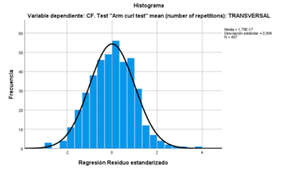	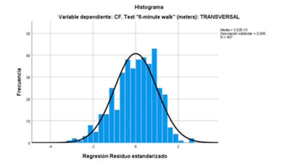	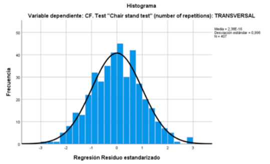	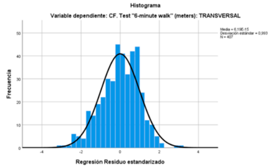	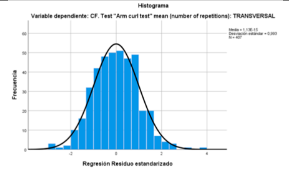
**Normal P–P plot of standardized residuals**	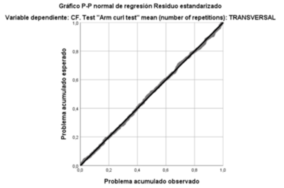	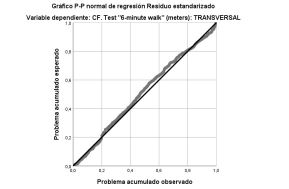	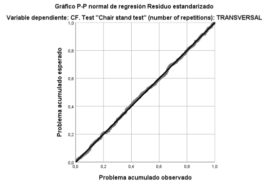	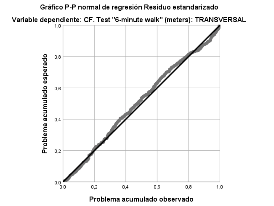	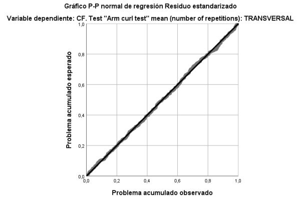
**Standardized residuals vs. standardized predicted values**	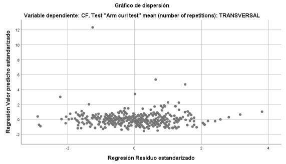	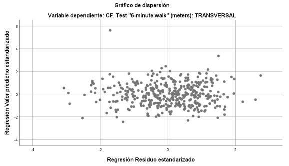	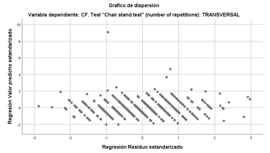	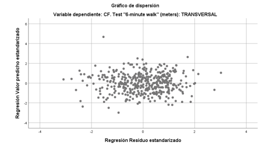	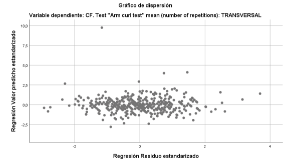

Diagnostic plots for the multiple linear regression models including vigorous physical activity as an independent variable. These plots are provided to facilitate the evaluation of the regression assumptions, given the highly skewed distribution of vigorous physical activity. For each model, the histogram of standardized residuals, the normal P–P plot of standardized residuals, and the scatterplot of standardized residuals versus standardized predicted values are presented. Visual inspection indicated no substantial deviations from the assumptions of normality, linearity, or homoscedasticity across all models.
